# Electron transport across the cell envelope via multiheme *c*-type cytochromes in *Geobacter sulfurreducens*

**DOI:** 10.3389/fchem.2025.1621274

**Published:** 2025-07-16

**Authors:** Media Zakizadeh Tabari, Allon I. Hochbaum

**Affiliations:** ^1^ Department of Chemical and Biomolecular Engineering, University of California, Irvine, Irvine, CA, United States; ^2^ Department of Materials Science and Engineering, Chemical and Biomolecular Engineering, Chemistry, Molecular Biology and Biochemistry, University of California, Irvine, Irvine, CA, United States

**Keywords:** electron transport, cytochrome nanowire, outer membrane cytochromes, geobacter sulfurreducens, extracellular electron transfer

## Abstract

Extracellular electron transfer (EET) enables certain microorganisms to respire using soluble and insoluble extracellular electron acceptors by transporting electrons across the cell envelope. Among these, *G. sulfurreducens* serves as a model organism for understanding direct EET pathways, where multiheme *c*-type cytochromes mediate electron transport from intracellular redox carriers to extracellular acceptors such as Fe(III) oxides and electrodes. This review focuses on heme-dependent electron transfer in *Geobacter sulfurreducens*, detailing the roles of inner membrane cytochromes, periplasmic carriers, outer membrane conduits, and recently characterized extracellular nanowires formed by polymerized multiheme *c*-type cytochromes, including OmcS, OmcE, and OmcZ. We examine the state of understanding of their physiological function, their structural features, expression patterns, and essentiality under various respiratory conditions. These insights advance our understanding of microbial anaerobic respiration and have implications for biogeochemical cycling, bioenergy generation, and bioremediation. The molecular architecture, assembly mechanisms, and secretion pathways of multiheme *c*-type cytochrome nanowires remain active areas of investigation, offering promising directions for future research and biotechnological innovation in engineered microbial systems.

## 1 Extracellular electron transfer: mechanisms and multidisciplinary relevance

Extracellular electron transfer (EET) is a general mechanism by which microorganisms transfer electrons across their cell membrane as part of their metabolic pathways. EET describes the processes by which organisms pass electrons between the intracellular and extracellular environments. EET plays a crucial role in biogeochemical cycling, particularly of iron and manganese in marine and terrestrial ecosystems (Shi et al., 2016; Tremblay et al., 2017). By modulating redox gradients, EET-active microbes influence sediment chemistry and microbial community structures, impacting carbon mineralization and anaerobic respiration. Beyond metal cycling, EET enables direct interspecies electron transfer (DIET), where electron-donating bacteria such as *Geobacter* spp. transfer electrons to syntrophic partners like methanogenic archaea, (Gao and Lu, 2021), or between *Geobacter* species to enable oxidation of diverse organic compounds (Summers et al., 2010). In addition to these roles, EET contributes significantly to plant and animal ecosystems by enhancing nutrient uptake in plant rhizospheres and influencing gut redox balance, host immunity, and pathogen colonization in animals (Stevens and Marco, 2023).

EET underpins biotechnological applications, including bioremediation, microbial fuel cells (MFCs), microbial electrosynthesis, metal recovery, and electric syntrophy. (Kato, 2015). In bioremediation, microbial EET reduces toxic metals such as Cr (VI) and U (VI) to insoluble forms, limiting their bioavailability and environmental impact (Wang and Shen, 1995; Lovley and Coates, 1997). MFCs exploit EET to convert organic substrates into electrical energy, enabling sustainable wastewater treatment and bioelectricity generation (Logan, 2009). Microbial electrosynthesis uses EET for CO_2_ fixation into value-added chemicals like acetate and biofuels (Kato, 2015). Additionally, EET enhances anaerobic ammonium oxidation (anammox), offering new strategies for nitrogen removal in wastewater treatment (Zhen et al., 2024). Expanding our understanding of microbial EET mechanisms holds great potential for advancing energy, environmental, and industrial biotechnology.

This review highlights recent advances in understanding heme-mediated EET mechanisms which enable an environmentally and technologically important form of anaerobic respiration. Using EET, anaerobic organisms can access extracellular electron acceptors, including soluble metal species, solid state minerals, or synthetic electrodes, as part of energy generating metabolic processes (Shi et al., 2016). EET occurs through proteins in direct contact with electron acceptors or donors, or via diffusible redox shuttles. We will survey the present understanding of the role of heme proteins, called cytochromes, in direct EET pathways employed primarily by the model organism, *Geobacter sulfurreducens*. These pathways encompass electron transfer from intracellular donors (e.g., menaquinone) to inner membrane, periplasmic, and outer membrane cytochromes and finally to extracellular electron acceptors.

## 2 *G. sulfurreducens*: a microbial platform for the study of extracellular respiration and cytochrome regulation

*Geobacter sulfurreducens* is a highly adaptive organism capable of respiring anaerobically on a range of terminal electron acceptors, both soluble and insoluble, in liquid cultures, as well as on solid state mineral and electrode surfaces relevant to microbial electrochemical technologies. The discovery and study of *G. sulfurreducens* as a model organism for EET were shaped by early studies that established its genetic tractability and unique respiratory capabilities. The development of a genetic system for *G. sulfurreducens* allowed researchers to introduce foreign DNA, create targeted gene mutants, and manipulate and develop reporters of gene expression, all critical steps in unraveling its EET pathways (Coppi Maddalena V. et al., 2001). Its genetic tractability, combined with foundational work demonstrating the organism’s ability to use electrodes as electron acceptors, cemented its role in the study of electrical current generation in MFCs (Bond and Lovley, 2003). Unlike organisms such as *S. oneidensis*, extracellular reduction of Fe(III) oxides by *G. sulfurreducens* requires direct contact between the organism and the insoluble electron acceptor (Nevin and Lovley, 2000). Early studies of dissimilatory metal reduction proposed the involvement of cytochromes in EET by the related bacterium *Geobacter metallireducens,* (Gorby and Lovley, 1991), and later work confirmed their requirement for the reduction of soluble (Leang et al., 2003; Lloyd et al., 2003; Butler et al., 2004) and insoluble (Mehta T. et al., 2005) electron acceptors by *G. sulfurreducens*. These findings placed the role of multiheme *c*-type cytochromes (MHCs) in shuttling electrons across cell membranes and to remote extracellular electron acceptors into sharp focus for this field of research in the following decades.

Another defining feature of *G. sulfurreducens* is its ability to form conductive biofilms that enable long-range electron transport and support dense packing of whole cell catalysts on anodes for microbial fuel cells. These biofilms maintain efficient electron transfer throughout the community to support anaerobic respiration several cell layers away from those in direct contact with the electrodes (Gorby and Lovley, 1991; Reguera et al., 2006). *G. sulfurreducens* enables this mode of growth by secreting a conductive biofilm matrix through which electrons shed by constituent cells access the oxidative potential of the anode surface. While the identity of the conductive elements that comprise this matrix was hotly debated, (Bond et al., 2012; Snider et al., 2012; Yates et al., 2016), these are now widely accepted to be networks of MHCs (see Section 5.2 for more details).

**FIGURE 1:**
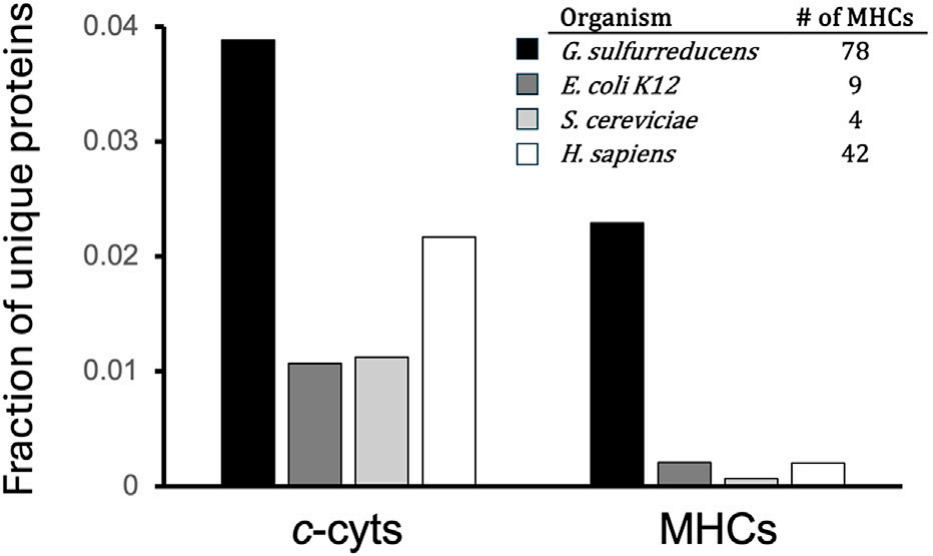
All (*c*-cyts) and multiheme (MHCs) *c*-type cytochromes as a fraction of total unique proteins encoded in the *G. sulfurreducens* genome as compared to other organisms. Inset: number of MHCs in *G. sulfurreducens* and other model organisms. Proteomes were downloaded June 2025 from UniProt (proteome ID *Gs*: UP000000577; *Ec*: UP000000625 *Sc*: UP000002311; *Hs*: UP000005640).

*Geobacter sulfurreducens* has become a model organism for studying the role of hemes in microbial anaerobic respiration. MHCs play a critical role in its adaptation and survival. Based on a search of its annotated genome for *c*-type heme binding motifs (CXXCH), *G. sulfurreducens* encodes 132 putative *c*-type cytochromes, 78 of which are MHCs (≥2 CXXCH motifs per protein). MHCs represent more than an order of magnitude greater fraction of the *G. sulfurreducens* proteome as compared to model microbes *Escherichia coli* (bacterium), *Saccharomyces cereviciae* (fungi/yeast), and even humans (*Homo sapiens*) (Figure 1). It is genetically tractable, exhibits numerous respiratory pathways that transport electrons into the extracellular environment, can grow as planktonic and biofilm phenotypes, and has varied contributions of MHCs to facilitating these respiratory pathways. Consequently, it provides fertile ground for studying a range of heme mediated electron transport phenomenon in microbial biology.

## 3 Where do the electrons come from? intracellular metabolic pathways generating electrons in *G. sulfurreducens*

In *G. sulfurreducens*, electrons are primarily generated through the complete oxidation of acetate via the tricarboxylic acid (TCA) cycle, producing NADH, NADPH, and reduced ferredoxin (Galushko and Schink, 2000; Mahadevan R. et al., 2006). Additional contributions can come from formate or hydrogen oxidation depending on growth conditions, but the TCA cycle remains the dominant source of reducing equivalents (Butler et al., 2006; Yang et al., 2010). Electrons derived from cytoplasmic metabolism, such as NADH oxidation, are transferred to the menaquinone (MK) pool via NADH dehydrogenase or alternative dehydrogenases, including formate and hydrogen dehydrogenases (Levar et al., 2017; Pimenta et al., 2023). NADH dehydrogenase (Complex I), for example, reduces menaquinone (MK) to menaquinol (MKH_2_) and transports protons across the inner membrane to generate a proton motive force (PMF). This is one of the few energy-conserving steps in *G. sulfurreducens*, and its continued function depends on maintaining an oxidized quinone pool (Meng et al., 2013).

The MK pool serves as an electron shuttle between cytoplasmic metabolism and membrane-bound electron transfer components, effectively linking intracellular respiration to periplasmic redox processes. Unlike many aerobic model organisms that use ubiquinone, *G. sulfurreducens* employs a menaquinone-based system more suited to anaerobic environments (Butler et al., 2006). Electrons from MKH_2_ are transferred to inner membrane-bound multiheme cytochromes such as ImcH and CbcL, which are selectively active under different redox conditions (Meng et al., 2013). These cytochromes relay electrons to a periplasmic pool of small triheme *c*-type cytochromes, notably PpcABCDE (Coppi et al., 2007).

These periplasmic cytochromes diffuse freely within the periplasm and serve as electron shuttles between the inner membrane and outer membrane conduits. While this segment of the pathway does not contribute further to PMF, it plays a critical role in redox homeostasis. Without effective electron disposal, the MK pool becomes over-reduced, stalling NADH oxidation and halting energy generation at Complex I (Meng et al., 2013). Constraint-based models identified redundant, parallel pathways for acetate assimilation and electron transport away from the MK pool that sustain electron transfer when specific enzymes are disrupted (Segura et al., 2008). This redundancy, along with energy-conserving proton translocation, ensures robust intracellular metabolism coupled to extracellular Fe(III) reduction (Mahadevan R. et al., 2006). This architecture—centered on cytochrome-mediated transfer across the cell envelope—is a hallmark of *G. sulfurreducens* physiology and enables respiration of using a wide range of extracellular electron acceptors.

*Geobacter sulfurreducens* also possesses several alternative pathways to achieve redox balance. FrdCAB is a bifunctional fumarate reductase/succinate dehydrogenase that *G. sulfurreducens* can use to reduce fumarate intracellularly as a terminal electron acceptor (Butler et al., 2006; Frühauf-Wyllie and Holtmann, 2022). *G. sulfurreducens* further enhances its metabolic flexibility by utilizing alternative substrates such as formate and hydrogen as electron donors, dumping electrons into the redox pool via formate dehydrogenase and hydrogenases (Rotaru et al., 2012). These reactions supplement NADH and ferredoxin generation under specific growth conditions. Metabolic flux analyses show that intracellular fluxes dynamically adjust to maintain redox balance depending on the available electron donors and acceptors (Yang et al., 2010).

**FIGURE 2:**
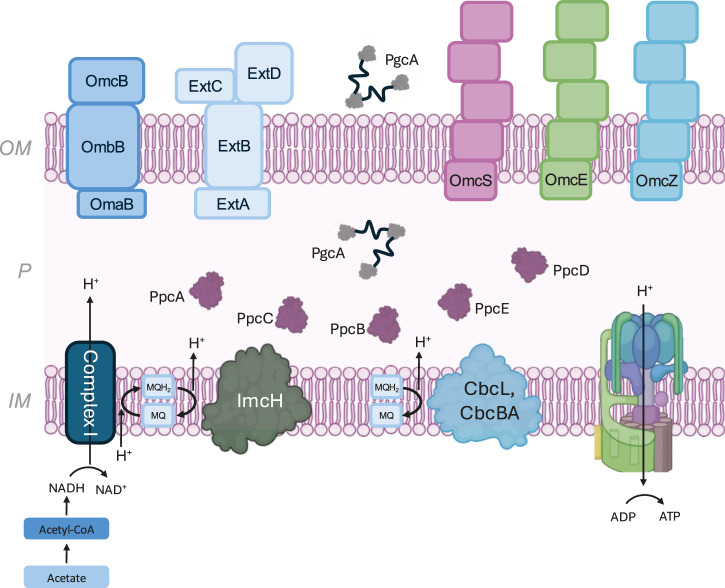
Components of the electron transfer pathway in *G. sulfurreducens*, from intracellular acetate oxidation to outer membrane and extracellular cytochromes. *IM* = inner membrane, *P* = periplasm, *OM* = outer membrane.

Overall, intracellular metabolism in *G. sulfurreducens* is optimized for electron production via acetate oxidation, with reducing equivalents entering the menaquinone-driven electron transport system (Figure 2). These steps link cytoplasmic metabolic pathways to electron transport pathways that end with the reduction of extracellular electron acceptors beyond the outer membrane. The presence of alternative metabolic pathways further enhances flexibility, allowing the organism to adapt to varying electron donor conditions.

## 4 How do electrons reach the outer membrane? electron transport across the periplasm

The electron transport in the *G. sulfurreducens* periplasm occurs through a set of complementary, sometimes redundant, and dynamic cytochromes and their redox interaction. Within the inner membrane, several key cytochromes regulate the oxidation of menaquinol (MKH_2_) and transfer electrons into the periplasm. The high-potential pathway involves the MHC ImcH, which oxidizes MKH_2_, releases protons into the periplasm, and transfers electrons to periplasmic cytochromes such as PpcA with high affinity (Levar et al., 2017; Joshi et al., 2021; Pimenta et al., 2023). ImcH functions efficiently at redox potentials above −0.1 V and shares structural features with the NapC/NirT family, including a conserved quinone-binding site (Levar et al., 2014; Joshi et al., 2021). In contrast, the *bc*-type cytochrome complex CbcL facilitates MK oxidation under low redox potential conditions, supporting respiration when high-potential pathways such as ImcH are inactive (Zacharoff et al., 2016; Joshi et al., 2021). Another cytochrome, CbcBA, is induced when more easily reduced electron acceptors are absent and participates in electron transfer at potentials near −0.2 V. This enables *G. sulfurreducens* to sustain respiration when energy availability is extremely low—conditions where most organisms would not survive—highlighting its ability to adapt to thermodynamically constrained environments (Joshi et al., 2021).

Electrons transferred from inner membrane cytochromes enter the periplasm through interactions with periplasmic MHCs. PpcA, a triheme MHC, serves as the primary electron shuttle within the periplasm, receiving electrons from ImcH and CbcL and facilitating their transfer to outer membrane electron transport systems (Lloyd et al., 2003; Antunes et al., 2022; Choi et al., 2022; Pimenta et al., 2023). The periplasmic cytochrome family (PpcA-E) compensates for the loss of individual members through functional redundancy, allowing electron transfer to proceed even when specific pathways are disrupted. Despite initial hypotheses suggesting specificity among these cytochromes for particular electron acceptors, deletion mutant studies indicate that multiple Ppc cytochromes compensate for each other, ensuring robust electron transfer in diverse environments (Choi et al., 2022).

Biochemical studies of PpcA-E show that these periplasmic electron carriers transiently interact with each other, transferring charge and equilibrating redox potential, resulting in a redundant and dynamic periplasmic electron transfer pool (Ferreira et al., 2024). Elimination of all five cytochromes abolished soluble Fe(III) citrate reduction, confirming the necessity of periplasmic electron transfer for linking inner membrane electron flow to the outer membrane conduits. Expression of PpcA alone partially restored Fe(III) reduction in a dose-dependent manner using an inducible promoter, further supporting the redundancy of PpcA-E and the capacity to reconstitute function through single components (Ueki et al., 2017). Similarly strains retaining any one Ppc paralog (ABCDE) were able to reduce Fe(III) citrate, indicating that each of these individual cytochromes were sufficient to support EET under those conditions and suggesting a lack of strict specialization (Choi et al., 2022). In contrast, when grown on electrodes as the terminal electron acceptor, a mutant lacking the periplasmic MHCs, PpcA-E, and PgcA, an unrelated MHC that also shuttles electrons across the periplasm, exhibited wild type-like current production and similar electrode-associated biomass, suggesting that other, as yet unidentified, electrode-specific pathways exist for periplasmic electron transport (Choi et al., 2022).

Beyond electron transport, periplasmic cytochromes contribute to energy conservation and efficiency. PpcA has been shown to exhibit redox-Bohr effects, which regulate proton coupling during electron transfer, optimizing bioenergetic efficiency (Silva et al., 2021). Additionally, transient binding interactions between inner membrane and periplasmic cytochromes, including those observed between PpcA and ImcH, (Pimenta et al., 2023), or CbcL, (Antunes et al., 2022), suggest that transient electron transfer complexes allow electrons to be passed quickly and efficiently between proteins without needing strong, permanent binding.

Modular electron transfer pathways extend from the inner membrane to periplasmic cytochrome interactions, allowing *G. sulfurreducens* to fine-tune its respiratory processes in response to environmental redox conditions (Figure 2). Comparative proteomic studies have revealed that cytochrome expression shifts dynamically in response to different electron donors, such as acetate or formate, highlighting metabolic adaptability (Mollaei et al., 2021). This ability to balance multiple electron transfer pathways through the periplasm while optimizing energy conservation is a key feature of *G. sulfurreducens* metabolism, enabling survival in diverse anaerobic environments.

## 5 From the outer membrane to the extracellular space: conductive appendages to access remote electron acceptors

### 5.1 Discovery of conductive bacterial appendages

The discovery of conductive bacterial appendages emerged from studies on dissimilatory metal-reducing bacteria, (Lovley, 1993), particularly *Geobacter*, *Shewanella*, and *Desulfovibrio* species, which can reduce extracellular insoluble acceptors such as Fe(III) oxides. In 2003, Bond and Lovley reported the first observations of EET from *G. sulfurreducens* to electrochemical cell anodes, (Bond and Lovley, 2003), and 2 years later, Reguera *et al.* provided the first evidence of the role of conductive microbial appendages in anaerobic respiration by *G. sulfurreducens* (Reguera et al., 2005). The latter study observed hair-like cellular appendages that appeared essential for reducing insoluble Fe(III) oxides. Conductive-probe atomic force microscopy (cp-AFM) revealed electronic conduction across the nanowire diameter (transverse) into an underlying graphite substrate, suggesting a physiological role as electron conduits (Reguera et al., 2005). Later studies confirmed the transverse (Veazey et al., 2011) and axial (Adhikari et al., 2016) (along their length) conductivity of individual *G. sulfurreducens* nanowires. Due to the high expression level of PilA, the diminished reduction of Fe(III) in a *pilA* deficient strain, and the fibrillar appearance of the presumed conductive appendages by TEM, researchers initially concluded these nanowires were *G. sulfurreducens* Type IV pili (T4P), (Reguera et al., 2005), which are commonly occurring appendages in many Gram-negative bacteria.

Parallel work on *Shewanella oneidensis* MR-1 also identified extracellular appendages as conductive components of *S. oneidensis* biofilms (Gorby et al., 2006; El-Naggar et al., 2010). These were later determined to be extensions of the outer membrane decorated with *MHC*s, such as MtrC and OmcA (Pirbadian et al., 2014; Subramanian et al., 2018). cp-AFM and scanning tunneling microscopy studies revealed discrete electronic states along these structures, attributed to redox-active hemes within these cytochromes, supporting a “superexchange” mechanism of charge transport, whereby electrons hop between cytochrome redox centers (El-Naggar et al., 2008; Strycharz-Glaven et al., 2011). Regardless of the physical structures supporting conductivity in these appendages, the conductive properties of these appendages have since been harnessed in bioelectrochemical applications, including microbial fuel cells, bioremediation technologies, environmental energy harvesting, and light-driven biocatalysis (Lovley, 2011; Logan et al., 2019; Liu et al., 2020; Neu et al., 2022).

### 5.2 Structure of conductive *G. sulfurreducens* appendages: bacterial MHC polymers

Early studies of *G. sulfurreducens* nanowires initially implicated Type IV pili (T4P) as the primary conduits for long-range EET from electrode- and Fe(III) particle-associated cells, and subsequent research over the next decade and a half appeared to support this model (Malvankar et al., 2011; Malvankar et al., 2015; Xiao et al., 2016). However, shortly after the discovery of conductive appendages in *G. sulfurreducens*, two cytochromes, OmcS and OmcE, were identified in extracellular preparations as essential for respiration via the reduction of insoluble Fe(III) oxides (Mehta T. et al., 2005). In a landmark study published in 2019, cryogenic transmission electron microscopy (cryo-EM) revealed a new structural model for these appendages, demonstrating that the conductive extracellular nanowires are, in fact, composed of polymerized OmcS (Wang et al., 2019). Evidence supporting this model and its reconciliation with previously published data have been extensively reviewed elsewhere (Wang et al., 2019; Wang et al., 2023; Yalcin and Malvankar, 2020; Gralnick and Bond, 2023).

The structure of extracellular OmcS nanowires is unique in several ways and provides a basis for understanding long range electronic conductivity in electrode- and metal oxide-associated cells. The nanowires are homopolymers of OmcS, a hexaheme *c-*type cytochrome, apparently secreted from the outer membrane, although the mechanism of secretion and the nature of the nanowire anchoring in the membrane are still speculative (Shen et al., 2025). OmcS nanowires arrange bis-histidine (His) coordinated heme in a one-dimensional chain along their axes with closely packed heme-heme distances varying from 3.4–6.1 Å (Wang et al., 2019). The OmcS subunits are attached by an extensive protein-protein interface and a metal coordination bond across this interface between the heme in one subunit and a distal His residue on the neighboring subunit. These structural features support a model for long range conductivity whereby electrons diffuse along the nanowires via successive reduction and oxidation of hemes in the axial chain.

Subsequent research has established that *G. sulfurreducens* produces at least three polymerized MHC appendages: OmcS, OmcE, (Wang et al., 2022b), and OmcZ (Yalcin and Malvankar, 2020; Wang et al., 2022a; Gu et al., 2023). Like OmcS, OmcE and OmcZ were previously identified as critical MHCs for the extracellular reduction of metal oxides and electrochemical anodes, (Nevin et al., 2009; Inoue et al., 2011; Franks et al., 2012), respectively. The functional similarities of these wires mirror the redundancy of periplasmic MHC electron transport pathways to adapt to changing redox conditions. Despite minimal sequence and structural similarity, OmcS and OmcE share a highly similar arrangement of hemes (Wang et al., 2022b). OmcS and OmcE are hexaheme and tetraheme *c*-type cytochromes, respectively, and both share the feature of inter-subunit coordination of one heme by a distal His across the subunit-subunit interface. The two polymers have a different helical rise, but hemes are arranged in both as linear chains of heme pairs that alternate between parallel and obtuse (“T-shaped”) angle packing of the porphyrin planes of neighboring hemes.

On the other hand, OmcZ, an octaheme *c*-type cytochrome, does not have inter-subunit heme coordination, and one of the eight heme in each subunit is offset from the axial chain and highly solvent exposed (Wang et al., 2022a; Gu et al., 2023). Nevertheless, OmcS, OmcE, and OmcZ nanowires all share the same axial parallel/T-shaped heme pair arrangement, apart from the two heme pairs in OmcZ that include the highly solvent exposed heme. Moreover, by plotting the angle of rotation between heme porphyrin planes and distances of nearest approach of heme porphyrin rings of heme pairs in these MHC polymers and in all MHC structures in the Protein Databank (PDB), it is evident that this heme pair geometry is largely conserved across all domains of life (Wang et al., 2022b; Baquero et al., 2023).

The secretion of MHC polymers is also not unique to *G. sulfurreducens*. Rather, it appears to be a broadly conserved appendage across domains of microbial life. Baquero et al. used bioinformatic screening and metagenomic analysis to identify candidate archaea that secrete polymerized MHCs (Baquero et al., 2023). Indeed, two candidate species, *Pyrobaculum calidifontis* and *Archaeoglobus veneficus*, produced MHC appendages, the structures of which were determined by cryo-EM. The heme chains in these archaeal appendages also arrange heme in pairs that fall within the distance and rotation angle clusters of most MHCs of the parallel and T-shaped geometries discussed above. Beyond experimentally determined structures, sequence homology searches have identified OmcS, OmcE, and OmcZ homologs across domains of bacteria and archaea, (Baquero et al., 2023; Gu et al., 2023; Portela et al., 2024), suggesting that MHC polymers represent a widespread microbial strategy for EET.

### 5.3 Physiological roles of MHC nanowires

#### 5.3.1 A note on challenges in studying the physiological roles of MHC nanowires

Determining the physiological roles of cytochrome nanowires in *G. sulfurreducens* presents significant experimental challenges. First, cytochrome expression and filament assembly are tightly regulated and condition-dependent. Mutating one cytochrome, such as OmcS, often leads to compensatory upregulation of others like OmcE or OmcZ, complicating interpretation of mutant phenotypes (Wang et al., 2022b). Second, filament assembly and secretion rely on proteins like the pilin building block, PilA-N, which are necessary for translocating OmcS and OmcZ to the extracellular environment. Deletion or modification of pilin genes disrupts this secretory process and alters the extracellular cytochrome profile, introducing secondary effects not directly related to nanowire function (Gu et al., 2021). For example, deletion of *pilA* in DIET cocultures of *G. sulfurreducens* and *Geobacter metallireducens* eliminated syntrophic growth, (Summers et al., 2010), but deletion instead of the pilin polymerization motor protein, *pilB*, maintained cytochrome secretion patterns and coculture growth (Liu et al., 2018). Third, different cytochromes have overlapping but context-specific roles—e.g., OmcZ is essential for anode respiration, whereas OmcS is more critical for Fe(III) oxide reduction—necessitating multi-gene knockouts and a range of growth conditions to fully resolve function (Mehta T. et al., 2005; Jiménez et al., 2018; Choi et al., 2022; Jiang et al., 2023). Another complication arises in electrical or imaging experiments: nanowires are often characterized in non-physiological conditions, making it difficult to (a) identify conclusively the cytochrome wire under test, and (b) to draw conclusions about their *in vivo* behavior (Wang et al., 2022b). Lastly, it is unknown whether OmcS, OmcE, and OmcZ are the only MHC appendages secreted by *G. sulfurreducens*, nor whether these cytochrome nanowires have electron transporting function in both their monomeric and polymerized forms. As a result, findings from studies conducted prior to the discovery of cytochrome-based nanowires, or those that do not characterize whether cytochromes are present in monomeric or polymeric form, should be interpreted with caution in light of these considerations.

#### 5.3.2 Functional insights from genetic studies

Gene knockout, complementation, and transcriptomic studies provide the broadest data set informing the physiological roles of cytochrome nanowires in EET. These studies provide evidence on how OmcS, OmcE, and OmcZ, the only *G. sulfurreducens* MHCs known as of the date of publication to form polymeric appendages, contribute to EET under different environmental conditions and electron acceptors. Evidence supports a model in which MHC nanowires are secreted by the cell (Figure 2) to respire using solid state electron acceptors, such as insoluble Fe(III) oxides and electrochemical anodes.

Gene knockout studies targeting key outer membrane cytochromes, including OmcS, OmcE, and OmcZ, demonstrate that single deletions typically do not abolish EET to insoluble Fe(III), but combinatorial deletions lead to impaired or abolished Fe(III) oxide reduction (Mehta T. et al., 2005; Holmes et al., 2006; Ammend et al., 2023). *omcS* and *omcE* are highly upregulated when *G. sulfurreducens* is grown on insoluble Fe(III) oxides, and single knockout mutants are deficient in Fe(III) oxide reduction except when soluble mediator are present in the growth medium (Mehta T. et al., 2005). OmcS is also essential for DIET between *G. sulfurreducens* and *G. metallireducens* under syntrophic growth conditions (Summers et al., 2010). *omcS* plays a role in early-stage growth on electrochemical anodes, but knocking out *omcE* appears to have little effect on current production from anode-associated communities (Jiang et al., 2023). However, while OmcS nanowires are isolated from cells grown on electrodes or in planktonic cultures with fumarate as a soluble electron acceptor, (Wang et al., 2019), OmcE nanowires are found in cultures of mutant strains deficient in OmcS, suggesting that OmcE nanowires may be secreted as a compensatory mechanism for the loss of OmcS under certain growth conditions (Wang et al., 2022b). OmcZ, on the other hand, is essential for growth on anodes (Nevin et al., 2009). It is highly upregulated during growth on anode surfaces, and *omcZ* deletion significantly inhibits current production. Immunolabeling indicates that OmcZ is found widely dispersed within the conductive matrix of *G. sulfurreducens* biofilms (Inoue et al., 2011). Correspondingly, growth on anodes stimulates the production of OmcZ nanowires, which are reported to have 100x greater electronic conductivity than OmcS nanowires (Yalcin et al., 2020).

In addition to the functional roles of MHC nanowires, deletion of five gene clusters in *G. sulfurreducens*, each encoding outer membrane electron conduit cytochromes and associated proteins, revealed that at least one is critical to electrode reduction (Jiménez et al., 2018). Deletion of the *extABCD* gene cluster severely diminished current production at electrodes, and complementation of only this cluster, and none of the four others, restored wild type current production. These results suggest a critical role for ExtABCD in electron transfer at the outer membrane, perhaps to nanowires like OmcZ which are highly upregulated when grown on anodes.

*Geobacter sulfurreducens* also employs outer membrane porin–cytochrome complexes (PCCs) for the reduction of other (not electrodes) electron acceptors. The OmbB–OmaB–OmcB complex, for example, has been proposed to bridge periplasmic electron carriers to external Fe(III) oxides and electrodes (Liu et al., 2014). Expression of other outer membrane complexes, such as ExtEFG and ExtHIJKL was similarly shown to be responsible for enhanced reduction of insoluble Fe(III) and Mn(IV) oxides (Jiménez et al., 2018). While genetic studies indicate that these PCCs play a role in EET, their involvement in cytochrome secretion remains speculative (Shi et al., 2014). It is currently unclear whether PCCs facilitate electron flow independently or if they act in conjunction with other extracellular electron carriers. Given the lack of direct biochemical evidence linking PCCs to nanowire formation, this model should be considered preliminary until further structural and functional studies provide additional validation.

Transposon mutagenesis has also been applied at the genome scale to uncover new genetic contributors to EET. In *G. sulfurreducens*, a genome-wide Tn-Seq analysis identified over 50 genes encoding cytochromes or related proteins whose disruption led to ≥50% reduction in current generation or Fe(III) respiration (Chan et al., 2017). These findings underscore the complexity of the EET network and identify candidate proteins for further targeted knockout studies.

These observations point to speculative physiological roles but also present more unanswered questions. For example, OmcZ is expressed predominantly when *G. sulfurreducens* biofilms are grown on poised anodes (Yalcin et al., 2020). It exhibits a highly solvent exposed heme–not present in OmcS or OmcE–that may play a role in creating a highly conductive nanowire mesh supporting thick biofilm growth by reducing inter-nanowire electron transfer barriers (Wang et al., 2022a). On the other hand, these nanowires evolved in the absence of oxidizing electrodes, there is no clear analog for these growth conditions in nature, and no environmentally relevant growth conditions have been identified to date in which OmcZ is as strongly and uniquely upregulated as on poised anodes. OmcS appears to be critical for DIET, (Summers et al., 2010), but has not been implicated in DIET beyond a synthetic consortium of *G. sulfurreducens* and *G. metallireducens*. It is possible that cytochrome nanowires are also key to DIET in natural consortia between *Geobacter* spp. and other species, such as in methanogenic cocultures with the archaeon *Methanosarcina barkeri*, (Rotaru et al., 2014), but such a connection has not been established. Even if it were determined that OmcZ and OmcS play distinct physiological roles, it remains unknown why *G. sulfurreducens* would maintain genes for both OmcS and OmcE, which share heme chain structural features, including poorly solvent accessible hemes (Wang et al., 2022a). OmcE and OmcS are similar in their role in the reduction of insoluble electron acceptors, (Mehta T. et al., 2005), and *G. metallireducens* OmcE (Gmet_2896) also facilitates DIET to *G. sulfurreducens* (Liu et al., 2018). We speculate that the complexity of cytochrome regulation and secretion have precluded a clear answer to this question. The use of cleaner background strains with all cytochrome nanowires deleted for genetic studies, in addition to well controlled electron acceptor conditions, will help shed light on this question.

### 5.4 Cytochrome nanowire assembly: models and unresolved questions

Early studies attributed *G. sulfurreducens* conductive filaments to T4P composed of PilA (Reguera et al., 2005). However, structural and genetic evidence now confirm that these filaments are instead polymerized MHCs, OmcS, OmcE, and OmcZ (Wang et al., 2019; Yalcin et al., 2020). The *G. sulfurreducens* T4P structure (Gu et al., 2021; Wang et al., 2022b) lacks the π-stacking of aromatic residues originally proposed to support conductivity and instead may function as a secretion apparatus rather than an electron conduit (Gu et al., 2021). The pilus differs from canonical T4P in both structure and function, resembling the pseudopilus of the Type II secretion system in *Escherichia coli*. Deletion of *pilA-N* abolishes the extracellular presence of OmcS and OmcZ (Gu et al., 2021). However, the exact mode of interaction between PilA and cytochromes remains unresolved, and alternative secretion pathways have not been fully excluded. While some studies propose that the pilus system of *G. sulfurreducens* is essential for cytochrome translocation, direct visualization of this process is lacking, and further work is needed to determine whether additional factors contribute to this mechanism. The existence of an alternative trans-periplasmic electron pathway independent of PpcA-E and PgcA during electrode-associated growth (Choi et al., 2022) could also indicate that OmcZ or other MHC nanowires may be anchored, or otherwise electronically interfaced, to the outer and inner membranes.

OmcZ nanowires may self-assemble extracellularly without the need for pilin-mediated secretion or porin complexes, indicating that alternative assembly mechanisms should also be considered (Gu et al., 2023). OmcZ requires proteolytic maturation before polymerization. OmcZ is initially produced as a 50 kDa long chain form (OmcZ_L), which must be cleaved by the serine protease OzpA to generate the 30 kDa short chain form, OmcZ_S, which is the repeat unit of assembled OmcZ nanowires (Gu et al., 2023). OzpA-deficient mutants fail to form OmcZ nanowires and show reduced EET, highlighting the necessity of this proteolytic step (Ayako et al., 2021). Structural analysis suggests that the bulky C-terminal domain of OmcZ_L sterically hinders filament formation, and cleavage by OzpA serves as a regulatory switch for nanowire biogenesis. Imaging suggests that OmcZ may self-assemble *in vitro* following proteolytic activation, implying that OmcZ nanowire assembly may be independently of pilin or porin-facilitated processes (Gu et al., 2023).

In summary, relatively little is currently known about the assembly and regulation of MHC nanowires. Studies that shed light on the protein structures and biosynthetic machinery involved in MHC nanowire assembly, their network of regulatory genes, and their physical mechanisms of anchoring and redox coupling in the cell membranes will be critical to addressing questions of their physiological roles in nature and overcoming technical challenges to their technological implementation.

### 5.5 Hemes on chains–a new paradigm for diffusible redox mediators?

Recent studies suggest a potentially new class of *G. sulfurreducens* cytochromes that may be involved in EET via diffusive electron transport. PgcA is a triheme *c*-type cytochrome in *G. sulfurreducens* which is secreted into the extracellular space and can support growth on insoluble electron acceptors (Smith et al., 2014). Transcriptomics show that *pgcA* is upregulated in growth conditions with insoluble Mg(IV) oxides as the terminal electron acceptor, (Aklujkar et al., 2013), and proteomics indicate that PgcA is more abundant in growth conditions with insoluble Fe(III) oxides as the terminal electron acceptor (Ding et al., 2008). Correspondingly, a *G. sulfurreducens ΔpgcA* mutant lacking this gene was incapable of reducing Fe(III) or Mn(IV) insoluble electron acceptors (Zacharoff et al., 2017). PgcA is distinct from many other MHCs in that each of the three hemes is bound to separate domains connected by extended (Pro-Thr)_n_ tethers (Fernandes et al., 2023). These tethers keep the three heme domains apart, allowing only transient contact between them. Recombinant PgcA can directly reduce Fe(III) and it may mediate diffusive electron transport from the outer membrane to remote electron acceptors, but probably only within a few dozen nm of the membrane surface (Nash et al., 2024). The flexible chain-like arrangement of PgcA heme domains suggest it can shuttle electrons using fewer hemes and less protein than extracellular wires, like OmcS, OmcE, and OmcZ. But the fact that it is not physically connected to the outer membrane surface means that it can be lost to other nearby cells or organisms, and the transient inter-domain collisions suggest that electron transfer would be slower than through rigid heme chains as in the wires. In the absence of PpcA-E, compensatory mutation of several key residues of PgcA results in its retention the periplasm, providing further evidence that it behaves as a diffusive mediator of electrons (Choi et al., 2022). Indeed, early proteomic analysis found abundant PgcA (GSU1761) in cytoplasmic fractions of *G. sulfurreducens* cultures, which could indicate that its localization depends strongly on the strain or growth conditions (Ding et al., 2006). The physiological role of PgcA is still unknown, but these studies suggest that, contrary to prior conclusions from the literature, *G. sulfurreducens* may have the capacity to perform EET with diffusive electron carriers, albeit over short distances.

## 6 Concluding remarks

Recent advances have transformed our understanding of EET, particularly in *G. sulfurreducens*, where polymerized multiheme *c*-type cytochromes (MHCs) have been established as the primary conductive appendages mediating long-range electron transport. The discovery that OmcS, OmcE, and OmcZ form polymerized nanowires, and the resolution of their molecular structures, has provided a mechanistic foundation for interpreting electron transport from the outer membrane to insoluble electron acceptors. Yet, significant questions remain regarding the physiological roles, regulation, and assembly mechanisms of these cytochrome filaments.

Key scientific challenges include determining the conditions under which specific MHC nanowires are produced and function, identifying their membrane anchoring and electron entry/exit points, and resolving whether monomeric and polymeric forms play distinct roles. The mechanisms underlying cytochrome secretion and nanowire biogenesis—whether mediated by pilins, porin complexes, or proteolytic processing—remain incompletely understood. Moreover, it is unclear whether *G. sulfurreducens* secretes additional, as-yet-uncharacterized nanowires, or to what extent its secretion mechanisms are conserved across diverse microbial taxa. The complexity of regulatory and functional redundancy among cytochrome families also complicates efforts to assign discrete roles to individual components.

Technological challenges persist as well. There is a need for improved *in situ* methods to identify and characterize MHC nanowires under environmentally relevant conditions, as well as strategies to manipulate their properties for bioelectronic applications. High-resolution imaging, redox potential mapping, and single-cell expression tools could help disentangle the complex, condition-dependent roles of individual cytochromes. A particularly promising area is the heterologous expression of MHC nanowires in industrially relevant host organisms, such as *E. coli* or *S. oneidensis*. Successfully reconstituting nanowire assembly and electron transfer in such chassis could enable scalable, tunable, and autonomously assembled bioelectronic devices. However, this effort will require coordinated expression of cytochrome maturation factors, biogenesis systems, regulatory proteases, and secretion machinery proteins—many of which remain poorly defined even in *G. sulfurreducens*.

Future research should prioritize mechanistic dissection of nanowire secretion and anchoring, systematic mapping of EET network redundancy under fluctuating redox conditions, and development of synthetic biology tools to engineer and control MHC assembly and electron flow. Addressing these challenges will not only resolve fundamental questions in microbial respiration but also unlock new possibilities in bioenergy, bioremediation, and engineered living electronics.
